# High RAC3 expression levels are required for induction and maintaining of cancer cell stemness

**DOI:** 10.18632/oncotarget.23635

**Published:** 2017-12-22

**Authors:** Laura C. Panelo, Mileni Soares Machado, María F. Rubio, Felipe Jaworski, Cecilia V. Alvarado, Leonardo A. Paz, Alejandro J. Urtreger, Elba Vazquez, Mónica A. Costas

**Affiliations:** ^1^ Laboratorio de Biología Moleculary Apoptosis, Instituto de Investigaciones Médicas Alfredo Lanari, IDIM-CONICET, Facultad de Medicina, Universidad de Buenos Aires, C1427ARO Buenos Aires, Argentina; ^2^ Laboratorio de Anatomía Patológica, Instituto de Investigaciones Médicas Alfredo Lanari, Facultad de Medicina, Universidad de Buenos Aires, C1427ARO Buenos Aires, Argentina; ^3^ Universidad de Buenos Aires, Instituto de Oncología “Angel H Roffo”, Area de Investigación, C1417DTB Buenos Aires, Argentina; ^4^ Laboratorio de Inflamación y Cancer, IQUIBICEN-CONICET, Facultad de Ciencias Exactas y Naturales, Universidad de Buenos Aires, Ciudad Universitaria, C1428EGA Buenos Aires, Argentina; ^5^ Argentine National Research Council (CONICET), C1425FQB Godoy Cruz (CABA), República Argentina

**Keywords:** RAC3, tumor, cancer stem cell, stem cells, mesenchymal cells

## Abstract

RAC3 is a transcription coactivator, usually overexpressed in several tumors and required to maintain the pluripotency in normal stem cells.

In this work we studied the association between RAC3 overexpression on cancer cell stemness and the capacity of this protein to induce cancer stem properties in non tumoral cells.

We performed *in vitro* and *in vivo* experiments using two strategies: by overexpressing RAC3 in the non tumoral cell line HEK293 and by silencing RAC3 in the human colorectal epithelial cell line HCT116 by transfection. Furthermore, we analysed public repository microarrays data from human colorectal tumors in different developmental stages. We found that RAC3 overexpression was mainly associated to CD133+ side-population of colon cancer cells and also to early and advanced stages of colon cancer, involving increased expression of mesenchymal and stem markers. In turn, RAC3 silencing induced diminished tumoral properties and cancer stem cells as determined by Hoechst efflux, tumorspheres and clonogenic growth, which correlated with decreased Nanog and OCT4 expression. In non tumoral cells, RAC3 overexpression induced tumoral transformation; mesenchymal phenotype and stem markers expression. Moreover, these transformed cells generated tumors *in vivo*.

Our results demonstrate that RAC3 is required for maintaining and induction of cancer cell stemness.

## INTRODUCTION

Tumorigenesis has been long known to resemble organogenesis and most tumors are heterogeneous containing many phenotypically and functionally different cells [1, 2]. In fact, this heterogeneity may be a consequence of the clonal expansion and selection from cells having mutations and genomic instability, as well as the evolution trough proliferation, maturation and differentiation from an original tumor initiating cell, called cancer stem cell [3]. Moreover, these mechanisms don’t have to be mutually exclusive and a combination of both could be contributing to the tumor development.

Whenever the case, what is really important to consider is that cancer stem cells side population is the major responsible of cancer propagation and resistance to most anti-cancer treatments. These cells have the ability to perpetuate the tumor when invade other tissues or when are transplanted, recapitulating the heterogeneity and histology of the parent tumor in the new focus.

Although there is a diversity of tumor types and treatments, most of them could be considered as targeting a systemic disease, where a definitive remission is usually not sure until several years after detection, surgery and/or treatment. Despite some cancer types are originally sensitive to chemotherapy, giving a promising response, after some years there is a tumoral relapse usually refractory to chemotherapeutic drugs. This could be explained as a the clonal selection of some resistant cells due to acquired mutations driven for the chemotherapeutic drugs and the self-patient physiology, as well as, the clonal expansion of cancer stem cells, originally resistant to chemotherapy. Whatever the way, the consequences are the same: human death by cancer.

These cancer stem cells have specific cell markers [4–6], although their origin and maintenance are not clear at present. They may alternatively be derived from stem cell pools, progenitor cells or differentiated cells that undergo trans-differentiation processes [2, 6].

RAC3 (also known as SRC-3, AIB1, ACTR, p/CIP, TRAM-1), originally identified as a nuclear receptor coactivator and member of the p160 nuclear receptor coactivator’s family [7], is now considered an oncogene [8–10]. Several evidences contribute to this new identity. Although it was firstly described as a molecule overexpressed in breast tumors [11], it was later discovered as an NF-κB coactivator [12] overexpressed in a broad spectrum of tumors [9, 13–15] and having additional cytoplasmic functions non related to their histone acetylase activity [16].

In fact, RAC3 is an oncogene that contributes to tumor development acting as a nuclear receptor coactivator of several transcription factors [12, 17] that control the expression of genes related to cell cycle progression and proliferation, inhibition of apoptosis [16, 18], autophagy [19] and senescence [20].

Additional functions related to tumor progression and metastasis development [17, 21], such as metalloproteinases expression, cell migration and epithelial-mesenchymal transition have also been described and attributed to the RAC3 splicing variant Delta 4-SRC3 [17, 22, 23].

Most of the studies that allowed to define RAC3 as an oncogene were performed in models where it is naturally overexpressed, such as tumoral cell lines, tumors and transgenic or knockout mice [8] where expression is equally affected in all the tissues. Although the transforming effect of RAC3 overexpression over non tumoral cells has not been deeply investigated up to date, we have previously demonstrated that RAC3 overexpression as a unique change, in the non tumoral human embryonic kidney cell line (HEK293) enables these cells to growth in soft agar forming colonies [24].

In addition, it was recently demonstrated that RAC3 expression is required in order to preserve the pluripotency and self-renewal of stem cells [25–28]. In normal healthy tissues RAC3 expression is downregulated in mature and differentiated cells, as well as in aged tissues [20], suggesting that changes in their levels may be playing a critical role in development. However, we have previously found that inflammatory cytokines like TNF up-regulate the expression of this oncogene [29].

Therefore, being RAC3 an oncogene and also a required factor to maintain the stem properties of normal cells, in this work, we investigated its association with cancer stem cell phenotype.

## RESULTS

### RAC3 overexpression contributes to maintain the mesenchymal phenotype

RAC3 is a known high expression molecule in several types of tumors including colorectal cancer [29]. However, it was not clearly determined at present if this overexpression could be early detected at tumor initiation and then homogeneously sustained along the cancer progression.

**Figure 1:**
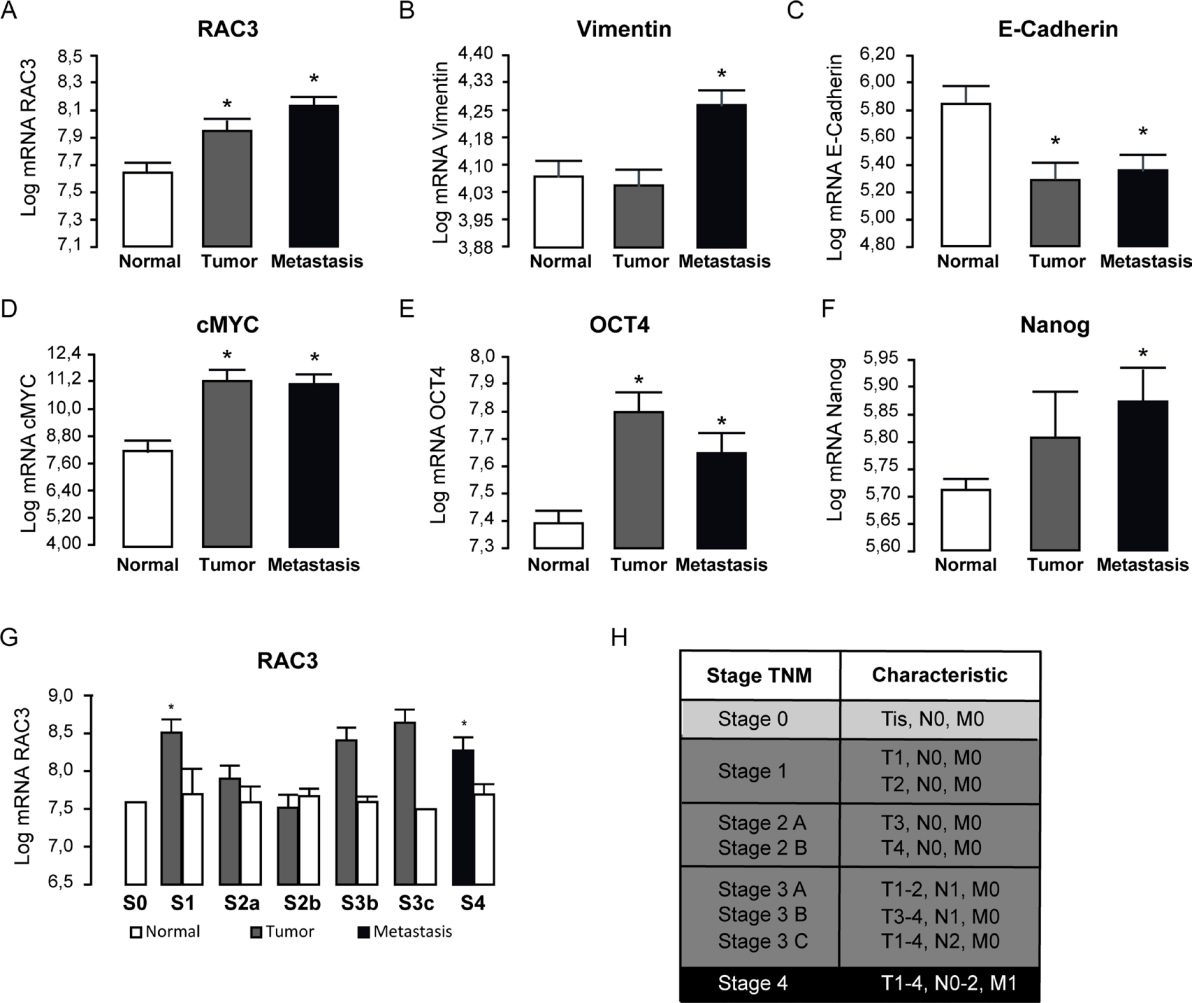
RAC3 overexpression and stem markers are mainly associated to the high expansion state of colorectal cancer in human patients The diagram bars shows the average ± SD Log_10_ expression levels of mRNA *(***A**–**G**). The stage (S) classification is shown in the table. T: tumor size; N: lymphatic ganglia propagation; M: methastasis (**H**) (E = Stage). (*T*-test, ^*^p 0.05; ^**^p 0,01). Platform GPL570 (Affymetrix Santa Clara, CA, USA), accession number GDS4718.

Therefore, we performed the analysis of microarrays from repository public data base of 19 human colorectal cancer samples. We found that RAC3 is a common elevated expression gene in this type of tumors and metastasis (Figure 1A), which correlated with an enhanced mesenchymal phenotype, increasing Vimentin and c-MYC, but decreasing E-Cadherin expression (Figure 1B–1D). Moreover, these changes were accompanied by an increased expression of stem cell markers OCT4 and Nanog (Figure 1E and 1F). Concerning cancer progression, RAC3 shows to be early overexpressed and maintained at advanced degree of the sickness (Figure 1G–1H).

**Figure 2:**
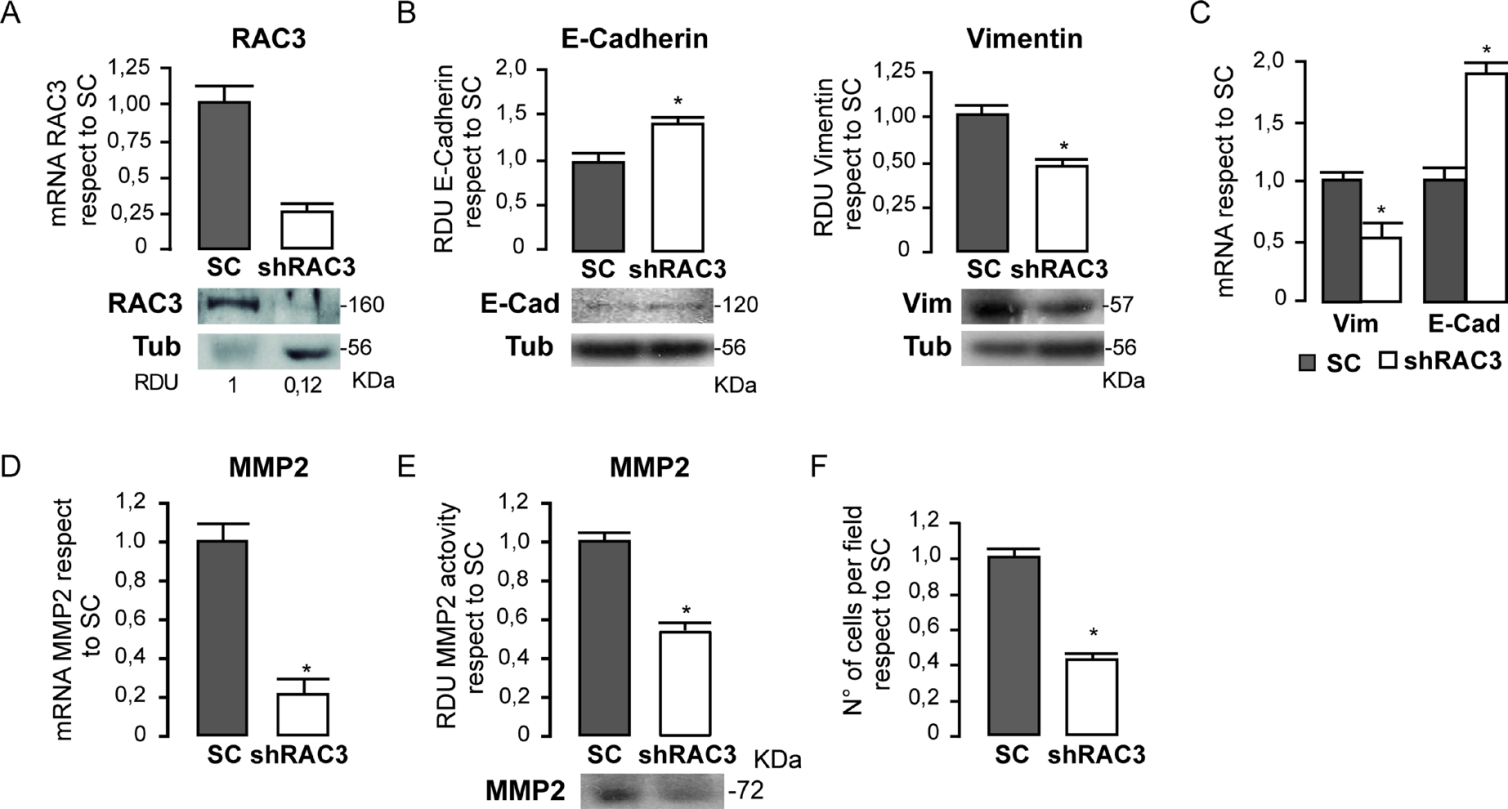
HCT116 colorectal cancer cells lose their mesenchymal properties when RAC3 expression is decreased The diagram bars shows the average ± SD expression levels of mRNA RAC3 in cells transfected with and shRAC3 or the scrambled (SC) carrying vector and the protein levels are shown in the lower panel (**A**). The diagram bars shows the mean ± SD of relative densitometric units (RDU) from three independent experiments of E-Cadherin and Vimentin in shRAC3 or SC (the lower panel shows a representative western blot) (**B**) or the mRNA expression levels determined by qPCR (**C**). Methaloprotease expression (**D**) and activity (**E**) are shown as the mean ± SD. Diagram bars shows the mean ± SD of invasive migrating cells (**F**).

In view of these results, we wanted to know if RAC3 could have a role inducing or maintaining some phenotypic properties of cancer stem cells that are shared with mesenchymal cells [30]. Therefore, we first investigated if a diminished expression of RAC3 may affect the expression pattern of Vimentin a typical marker of tumoral migratory, mesenchymal and some endothelial cells [31] and the epithelial marker E- Cadherin. In these experiments we compared the levels of both mRNA and proteins in the human cancer colon HCT116 cell line having high expression levels of RAC3 [19], stably expressing an scrambled or the shRAC3 expression vector, which significantly inhibits the RAC3 expression as shown in the Figure 2A.

We found that cells having low RAC3 expression shown an increased expression of E- Cadherin while Vimentin was downregulated respect to the control cells. Thus, these results demonstrate that RAC3 inhibition induces a mesenchymal-epithelial transition (MET) (Figure 2B and 2C).

In addition, MET induced by RAC3 inhibition was accompanied by a decrease in the metaloprotease-2 (MMP-2) activity and expression, as shown in Figure 2D and 2E, that also correlated with a diminished migratory and invasive behavior respect to control cells (Figure 2F). All these effects of MET, MMP- 2 downregulation and migratory-invasive behavior inhibition, were also obtained in the human mammary cancer T47-D cells where its natural high RAC3 expression was stably inhibited by an shRAC3 RNA (data not shown), demonstrating that is not exclusive of colon cancer.

### RAC3 overexpression contributes to maintain the cancer stem cell side population of colorectal cancer cells

Regarding the previously reported property of cancer stem cell population to quickly exclude the Hoechst staining through their high ABC-MDR expression levels [32], we performed experiments to detect differences in the stem cell amount between cells overexpressing or not RAC3 in the absence or presence of Verapamile in order to block the ABC transporters activity.

**Figure 3:**
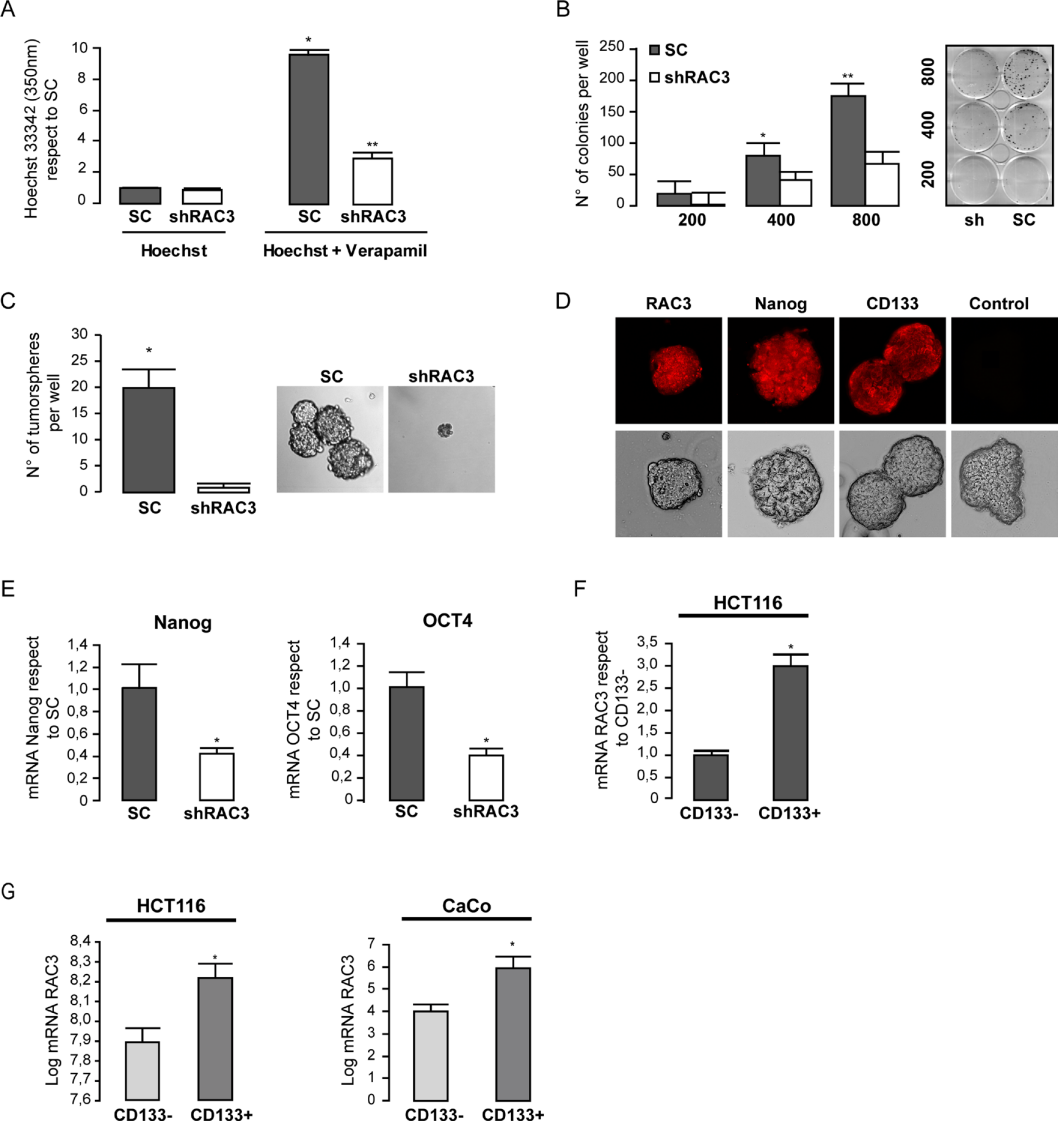
Colorectal cancer cells require high expression levels of RAC3 in order to maintain some cancer stem properties The diagram bars shows the values of intracellular Hoechst determined by spectrophotometry at 350 nm in HCT116 (shRAC3/SC) in the presence or the absence of Verapamil. (^*^p < 0,01, respect to Hoechst SC; *t*-test) (^**^p < 0.05, respect to Hoechst Sh; *t*-test). (**A**)**.**The diagram bars shows the mean ± SD of HCT116 colonies growth each one from one clone, when cells were plated at densities of 200, 400 or 800 per well (**B**)**.** Only SC cells were capable to growth as tumorspheres in stem media. Left panel shows the mean ± SD and (a representative image at right panel) (**C**). High RAC3 and stem markers detected by IFI in tumorspheres from SC-HCT116 using the specific primary antibodies or unspecific control α-mouse 594 (**D**). Expression levels of mRNA stem markers were determined in SC or shRAC3–HCT116 cells (mean from three independent experiments ± SD) (**E**). The diagram bars shows the mean ± SD mRNA RAC3 expression levels in HCT116 cells from CD133 enriched (CD133+) or depleted (CD133-) side population (**F**). Similar to F, but from both public repository data microarrays of FACS sorted HCT116 cells and CaCo cells (^*^p < 0.05 respect to CD133-). Platform GPL6244, accession number GSM932995 for HCT116 (colon cancer cells). And platform GPL96, accession number GSE24747 for CaCo (colon cancer cells), both from Affymetrix Santa Clara, CA, USA. The side population CD133+ o CD133− isolated by flow cytometry (**G**).

We found that in the absence of the blocking drug no differences in the amount of cells retaining the stain over 90 minutes were observed between cultures expressing high or low levels of RAC3 (Figure 3A). However, when the ABC transporters were blocked, although the total stain retention was increased, the amount of unstained cells was significantly inhibited in shRAC3 cells respect to control, demonstrating a decrease of cancer stem cell population in cells having low expression levels of RAC3.

In order to confirm these results by additional assays, we determined the clonogenic growth of control and shRAC3 HCT116 cells after seven days of platting at low density. As shown in Figure 3B, the number of colonies was significantly diminished in cells where RAC3 was inhibited. Moreover, cells expressing low RAC3 levels were almost unable to growth as tumorspheres (Figure 3C). This was in agreement with a diminished amount of cancer stem cells markers OCT4 and Nanog [33] (Figure 3E), while a high expression of the colon cancer stem cell marker CD133 and Nanog were detected by immunofluorescence staining of tumorspheres, as expected for their high amount of stem cells (Figure 3D).

We then analyzed if the expression levels of stem markers could be also affected by a decreased RAC3 expression in HCT116 cells. Figure 3E shows that expression of both Nanog and OCT4 were downregulated under low RAC3 levels.

In order to determine if RAC3 overexpression could be mainly associated to one cancer stem cell marker, we first isolated the CD133+ enriched side population from wild type HCT116 cells using a specific antibody conjugated to magnetic beads. The non-retained fraction was considered as CD133− side-population. Then, the expression levels of RAC3 mRNA were measured in both fractions. As shown in Figure 3F, high expression levels of RAC3 were mainly associated to the CD133 positive side population. Similar results were obtained when we processed the public repository microarrays data from HCT116 cells and the human epithelial colorectal adenocarcinoma CaCo cells (Figure 3G, left and right panels, respectively).

Taken together all these results, we may conclude that RAC3 overexpression is mainly associated to colorectal cancer stem cell markers, and contributes to maintain some cancer stem properties.

### RAC3 overexpression in non tumoral cells induces a mesenchymal transformation

In view of the results obtained in tumoral cells that naturally overexpress RAC3; we first investigated if RAC3 overexpression in non tumoral cells as a unique genetic introduced modification, is able to induce some of the tumoral properties that were observed in the human colorectal cancer cells.

**Figure 4:**
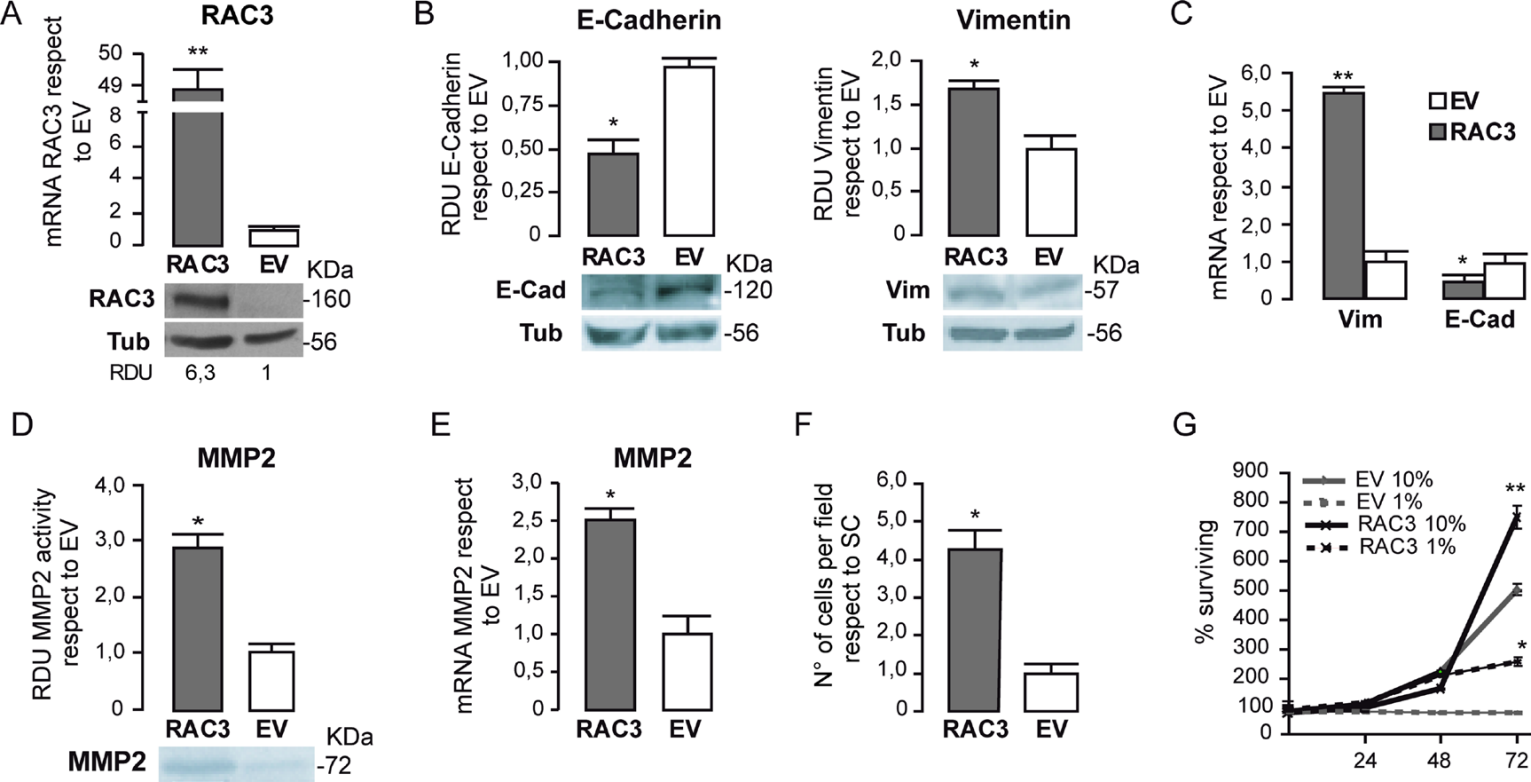
RAC3 overexpression in non tumoral cells induces a mesenchymal phenotype The diagram bars represents the mean ± SD of RAC3 mRNA expression levels in HEK293 cells respect to control EV and the protein levels are shown in the lower panel (**A**). The diagram bars shows the mean ± SD of RDU respect to control (EV) (**B**) or mRNA levels (**C**) from HEK 293 cells, stably transfected with an empty (EV) or RAC3 vector (RAC3). The diagram bars is the mean ± SD of MMP activity RDU respect to EV (**D**) or mRNA expression levels (**E**). Invasion assay of HEK293 cells overexpressing or not RAC3. The diagram bars shows the mean ± SD of cell migrating number respect to control EV (**F**). Cell surviving curve of HEK293 overexpressing (RAC3) or not (EV) the RAC3 growing in low serum (1% FBS) or in normal serum condition (10% FBS) at 24, 48 and 72 h (^**^p < 0.01, respect to control EV 10% or EV 1%) (**G**). (F). (^*^p < 0.05; ^**^p < 0.01; respect to control, *T*-test).

The non tumoral HEK293 cells express limited amounts of this coactivator, similar to normal mature differentiated cells [16]. They were stably transfected with a RAC3 expression vector, thus, significantly increasing the RAC3 levels respect to the cells carrying the empty vector as shown in Figure 4A.

We found that Vimentin levels were significantly increased while E-Cadherin levels were inhibited in cells overexpressing RAC3 (Figure 4B and 4C), supporting the results obtained in tumoral cells and the role of RAC3 overexpression favoring the mesenchymal phenotype.

In addition, this transition to a mesenchymal phenotype when RAC3 was overexpressed was accompanied with an increased activity of MMP-2 as determined by zymography (Figure 4D), which correlated with the enhanced MMP-2 gene expression (Figure 4E) and acquired migratory and invasive behavior. Although control HEK293 cells were almost unable to migrate and invade, we found they acquired this ability under RAC3 overexpression (Figure 4F).

We then investigated whether RAC3 overexpression could confer to these non tumoral cells the ability to proliferate in the presence of low growth factors. Therefore, the cell number was determined daily during three days in cultures of HEK293 overexpressing RAC3 or controls growing under normal or low serum (1%) conditions. Although control cells were unable to proliferate under low serum, RAC3 overexpressing cells acquired this ability (Figure 4G) increasing the cell number during 72 h of culture respect to control cells.

### RAC3 overexpression in non tumoral cells induces tumor initiating cells

**Figure 5:**
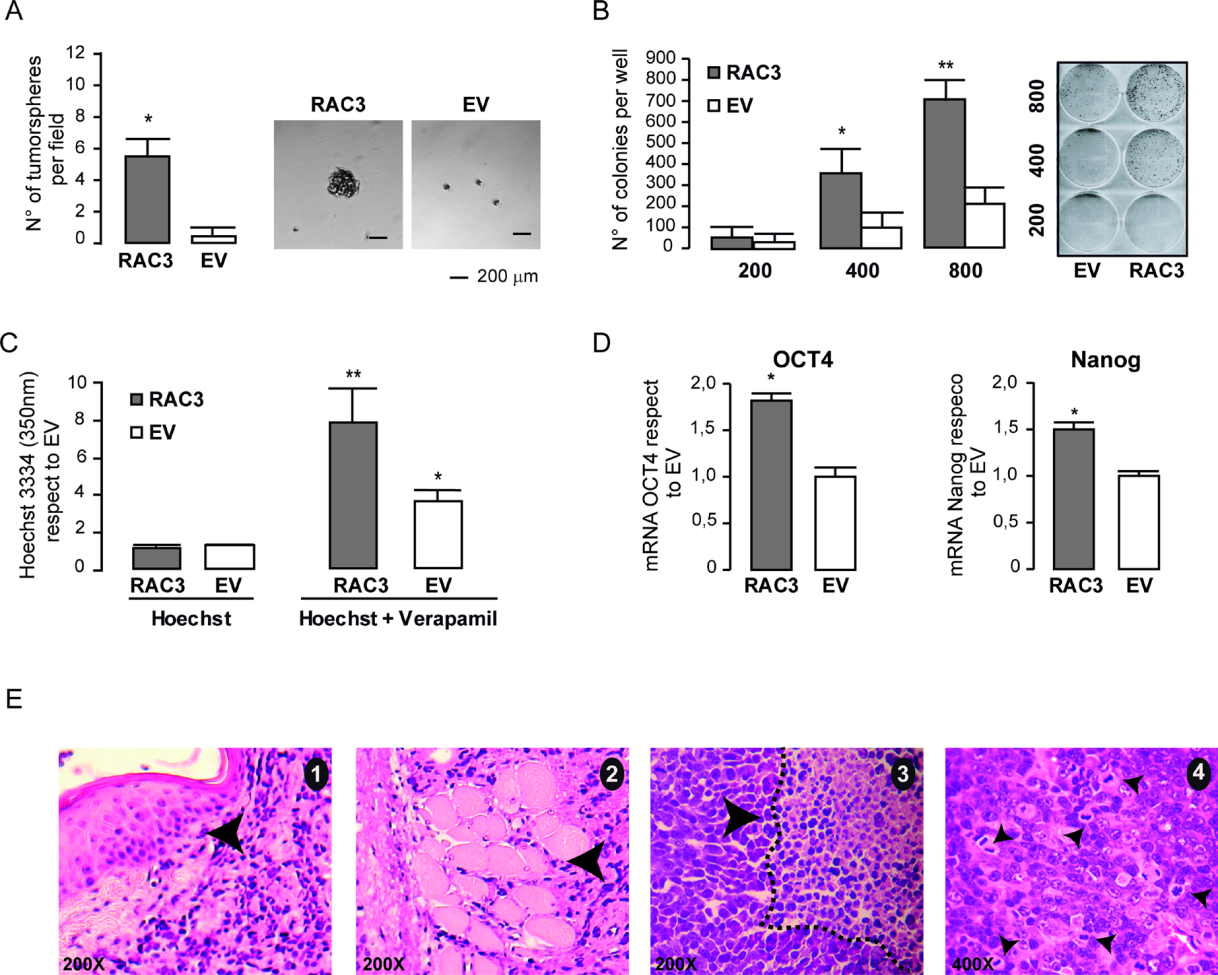
RAC3 overexpression induces a cancer stem like phenotype The diagram bars represents the mean ± SD of the tumorsphere number (left panel), while the right panel shows the tumorsphere morphology in HEK293 (RAC3 or EV) (**A**). The diagram bars represents the mean ± SD (left panel) of the colony number (^**^p < 0,01 respect to EV: ^*^p < 0,05 respect to EV) (**B**). The diagram bars shows the values of intracellular Hoechst determined by spectrophotometry at 350 nm in HEK293 (EV/RAC3) in the presence or the absence of Verapamil. (^**^p < 0.01, respect to Hoechst RAC3. *T*-test) (^*^p < 0.05, respect to Hoechst EV, *T*-test) (**C**). The diagram bars shows the mean ± SD of Nanog and OCT4 mRNA from HEK293 overexpressing (RAC3) or not (EV) RAC3 (^*^p < 0.05 respect to control; *T*-test (**D**). Tumor histology shows dermal infiltration (black arrow) and skeletal muscle infiltration (arrowhead) by tumor. Pictures 1 and 2, H&E 200 X magnification. The tumor necrosis (dotted line) and non-necrotic area, picture 3 H&E 200X. Atypical neoproliferation with numerous anomalous mitoses, picture 4, H&E 400X (**E**).

In order to investigate if this increase in cell number could be due in part to the enhanced stem cell side population in cultures overexpressing RAC3, we first investigated the ability of these cells to growth as tumorspheres. We found that although control HEK293 were almost unable to growth as tumorspheres, they acquired this ability under RAC3 overexpression (Figure 5A), while the clonogenic growth was significantly increased (Figure 5B). Moreover, experiments of Hoechst efflux (Figure 5C) also demonstrate the increase of stem cells in cells overexpressing RAC3. Therefore, we analyzed the expression levels of the stem cell marker genes Nanog and OCT4. As shown in Figure 5D, mRNA levels of both were significantly increased by RAC3 overexpression.

Our findings demonstrated that RAC3 overexpression as the unique introduced genetic modification in non tumoral cells, induced a cell transformation toward a phenotype that suggests the presence of cancer stem cells.

Therefore, we then investigated whether these cells were capable to initiate tumors when they are inoculated *in vivo*.

Although the wild type or empty vector transfected cells were unable to induce tumors, two mice inoculated with HEK293 overexpressing RAC3 developed tumors that after 18 days of initiation acquired a size of 5,8 and 6,8 mm. The hystopathological analysis of sections from these samples, identified them as malignant tumors showing high mitosis, some necrosis, angiogenesis and invasion of the skeletal muscle (Figure 5D).

## DISCUSSION

RAC3 is an oncogene having nuclear and cytoplasmic functions usually overexpressed in several tumor types, but having very low or undetectable expression levels in normal mature differentiated cells. Moreover, a few years ago it was also demonstrated that stem cells require RAC3 expression in order to maintain the pluripotency [25–28]. Likewise, it is downregulated when stem cells are differentiated in a similar way that the other stem gene markers [33]. The fact that RAC3 is a molecule whose detection at high or moderate levels could only be found in normal stem or tumoral cells open the intriguing question regarding a possible link between them.

Although its probably association to cancer stem cells has not been previously investigated, in this work we demonstrated that RAC3 overexpression is mainly associated to a cancer stem marker enriched side population and is required to maintain some cancer stem properties of colorectal cancer cells.

The original definition of cancer stem cells comes from the functional evidences concerning the ability to regenerate the tumor. Furthermore, the identification of specific markers associated to different tumor types was used to select side populations enriched in cancer stem cells, such as CD44 and CD133 [5, 34]. The assays performed with cancer stem cells enriched populations obtained by specific marker selection or limit dilution from the parental tumor allowed to deeply investigate their properties and behavior. In addition to their ability for tumor regeneration and *in vitro* growing as spheroids [35], these cells are the most resistant to chemotherapeutic drugs, being the high MDR expression at least one of the reasons [32, 36], in agreement with our previous works demonstrating that cells overexpressing RAC3 are more resistant to apoptosis, senescence and autophagy [16, 18–20]. Therefore, integrating all our previous and present results it is not surprising a probably association of RAC3 overexpression with a cancer stem cells.

In this work we demonstrated that although RAC3 is a high expression molecule in human colorectal tumors, its levels could be modulated along the cancer progression together with cancer stem cell markers, being probably higher in states associated with initiation, major propagation invading secondary focus, and aggressiveness where an important amount of cancer stem cells are required, having the ability to migrate and invade new tissues in order to build the new metastatic focus.

Several evidences support the oncogenic role of RAC3 [9, 13–15], and being RAC3 a NF-kB coactivator, and Vimentin a target of this transcription factor [24], its contribution to maintain the mesenchymal phenotype could be not surprising. However, their tumoral transforming effects when is overexpressed in non tumoral cells has not been previously investigated.

It was previously demonstrated that both RAC3 or the splicing Delta 4-SRC3 variant downregulation inhibits the oncogenic potential of cancer cells affecting several pathways related to its activity as a coactivator of nuclear receptors or the cytoplasmic signaling [23].

In general the genetic modification of a unique oncogene is not strong enough to induce a deployment of tumoral characteristics, but here we reported that RAC3 overexpression in non tumoral cells induces the cell transformation acquiring a typical tumoral phenotype with the concomitant ability to migrate, to invade, to produce metalloproteinases, to growth as spheroids even under low serum, to increase the expression of stem cell markers [3, 5, 33, 35] and the most strong evidence of the presence of cancer stem cells, like the ability to initiate tumors *in vivo* showing malignant hystopathological diagnose.

Because our studies of overexpression were performed in non tumoral cell lines but not in normal primary tissues, we cannot exclude that chromosomic alterations and the immortality condition could be contributing to the RAC3 transforming effect, giving a permissive signaling context. In addition, independently of the oncogenic profile, is the association of cancer stem markers and properties with RAC3 overexpression, which was supported through our findings analyzing microarrays from tumoral tissues and cell lines. Although these RAC3 overexpressing cells induced a rapid tumor growth when were inoculated *in vivo* in *nude* mice, their ability to be differentiated toward other several tissues was not demonstrated in the present work, afterwards, their classification as “stem cells” is not completely suitable.

Concerning the origin of cancer stem cells is currently unclear. It has been suggested that cancer can be considered as a disease of unregulated self-renewal in which mutations convert normal stem cell self-renewal pathways into engines for neoplastic proliferation [37, 38]. In this regard, there are evidences that support this origin, as the expression of some common stem marker genes [5, 34, 35, 39, 40]. However, there are not evidences that avoid to excluding the origin of cancer stem cells from mature differentiated cells carrying accumulated mutations or epigenetic changes.

Although the mechanism by which RAC3 is overexpressed, as a conserved signal in such amount of tumors remains unclear, it is possible to speculate this is a cancer stem cell condition, then heritable to the heterogeneous tumoral progeny. Moreover, an additional intriguing question still not solved arises from this hypothesis, concerning the origin of cancer stem cells. They can be a mutated product from normal stem cells, but the possible origin from normal differentiated mature cells acquiring mutations and epygenomic changes could not be excluded. In the first case, the increased RAC3 expression could be a heritable marker from the normal stem cell that once mutates to give a cancer stem cell, but in the second, we have to consider the possible signaling inducing this overexpression in mature differentiated cells. In this regard, inflammation is increasingly recognized as a critical component in tumor initiation and progression [41, 42]. Furthermore, we have previously demonstrated that inflammatory cytokines as TNF may upregulate the RAC3 gene expression [29]. Therefore, in this context, the induction of malignant or premalignant lesions from cells acquiring this RAC3 overexpression and possible additional mutations or epygenomic rearrangements sounds as a possible source for tumor initiating cells.

Finally, as a concluding remark, our results demonstrate that non tumoral cells may acquire a cancer stem like phenotype when RAC3 is overexpressed, suggesting that this switch deserves to be investigated *in vivo*, being a possible early step in tumor initiation when it occurs in normal differentiated cells having or not accumulated additional mutations. Although the cancer stem cells origin is still unclear, our results demonstrate that the phenotype of cells where RAC3 is overexpressed resembles some characteristics of normal stem cells, like the expression of stem gene markers and growing behaviour. Although the requirement of RAC3 expression for normal stem cells has been demonstrated, the consequences of overexpression over them, remains as an intriguing question to be investigated in order to improve the strategies for anti-cancer treatments and also that involving stem cell therapies.

## MATERIALS AND METHODS

### Cell culture and reagents

The non-tumoral cell line human embryonic kidney HEK293, was maintained in Dulbecco’s Modified Eagle Medium (DMEM) high glucose and the tumoral cell line HCT116 (human colon cancer) was maintained in DMEM F12 (Gibco Laboratories, Grand Island, NY, USA). All cultures were supplemented with 10% fetal bovine serum (FBS) (Invitrogen), penicillin (100 U/ml) and streptomycin (100 µg/ml). Cells were maintained at 37°C in a humidified atmosphere with 5% CO_2_. The HEK293 and HCT116 cell lines were acquired from ATCC. All the cell lines are usually tested for mycoplasma contamination once a month.

HEK293 cells were transfected by the CaCl2 method and HCT116 cells using Lipofectamine 2000 (Invitrogen).

Unless stated otherwise, all reagents were obtained from Sigma Chemical co. Bs. As., Argentine or Santa Cruz Biotechnology, USA.

### Expression vectors and reporter plasmids

HEK293 was transfected with an expression vector for RAC3 (pCMV-Tag 2B-RAC3) or with the empty vector (EV) as control (Invitrogen) and in the case of HEK293, selected for stable expression with Neomycin. HCT116 cells were transfected with an expression vector for shRNA-RAC3 (pRV-GFP-puromycin) or the scramble control, as previously described in our laboratory and selected for stable expression with puromycin [19].

Once transfected and after selection of cells for stable expression, the cultures were amplified and preserved by frozen in liquid nitrogen. After defrost and before to each experiment the RAC3 expression levels were tested using qPCR and western blot and no more than teen passages were used in order to ensure the original heterogeneity along passages.

### Inmunofluorescence

Immunofluorescence assays were performed as previously described [16]. Briefly, HCT tumorspheres were fixed with Glutaraldehyde 1% and formaldehyde 0,2%, permeabilized with PBS-Triton 0.2%, blocked with 10% FSB and incubated 2 h at room temperature with 0,5 µg/ml of the following antibodies: Nanog (sc: 293121), RAC3 (sc:25742) (Santa Cruz Biotechnology) and CD133 (AC133) (Milteni). Finally tumorsphere were incubated with fluorochrome conjugated secondary antibodies and visualized with a fluorescence microscope Olympus BX51 and photographed at 100× magnification.

### Western blot analysis

Western blot assays were performed as previously described [29]. Briefly, monolayers were scraped and treated with RIPA buffer containing pepstatin A, phenymethylsulfoyl fluoride and dithiothreitol, solubilized in SDS-PAGE sample buffer, separated on 8% SDS-PAGE, and electro-transferred to a nitrocellulose membrane.

Membranes were blocked for nonspecific binding with TBS 5% milk and 0.05% Tween-20 (T-TBS) and incubated overnight in T-TBS/0.5% BSA with 0.05 µg/ml of anti-RAC3, anti-Vimentin or anti-E-Cadherin, primary antibodies. Subsequently, membranes were washed and incubated for 1 h with HRP-conjugated secondary antibody, developed by chemiluminescence (Santa Cruz Biotechnology).

### PCR and qPCR assay

PCR and qPCR assay was performed as previously described [29]. Briefly, total RNA was isolated from HEK293 and HCT116, transfectants expression RAC3 or not. RNA Extraction was performed by using the TRIzol protocol (Invitrogen). Reverse transcription was carried out by using the SuperScript II kit (Invitrogen) following the manufacturer’s instructions. The gene expression analysis was performed by using sequence-specific primers for:

hRAC3 forward 5′-aagtgaagagggatctgga- 3′ and reverse 5′-cagatgactaccatttgagg-3′, CD133 forward 5′- cactaccaaggacaaggcgttc-3′ and reverse 5′-caacgcctctttggt ctccttg-3′, NANOG forward 5′-ctccaacatcctgaacctcagc-3′ and reverse 5′-cgtcacaccattgctattcttcg-3′, OCT4 forward 5′- cctgaagcagaagaggatcacc-3′ and reverse 5′-aaagcggcag atggtcgtttgg-3′, Vimentin forward 5′-gaacctgagggaaactaat ctg-3′ and reverse 5′-ctgagaagtttcgttgataacc-3′, E-Cadherin forward 5′-tggtcaaagagcccttactg-3′ and reverse 5′-caagtcaaa gtcctggtcct-3′, MMP2 forward 5′-ccagaataccatcgagacca-3′ and reverse 5′-gtagccaatgatcctgtatgtg-3′, GADPH foward 5′-tctcctctgacttcaacagc-3′ and reverse 5′-gttgtcatacc aggaaatga-3′ was used as an internal control.

### Clonogenic assay

Clonal cell growth was performed as previously described [43]. Briefly, single-cell suspensions containing 200–800 cells were seeded onto 6 well plates in MEM with 10% of FCS. Medium was changed every 72 h. After 7 days of culture the number of colonies was determined using an inverted microscope.

### Tumorspheres

The tumorspheres assay was performed as previously reported [44]. Briefly, 5 × 10^4^ HEK293 RAC3 or EV and HCT116 SC or shRAC3 cells were maintained in DMEM supplemented with B27 (Stem Cell Supplemented Invitrogen), EGF 20 ng/ml (Invitrogen) and penicillin/streptomycin in six well plates pre-coated with 1% agarose. Cells were maintained at 37°C in a humidified atmosphere with 5% CO_2_. The presence of tumorspheres was evaluated daily using light microscope and its number determined after 14 days.

### Invasion assay

The invasion assay was performed as previously described [45]. Briefly, 5 × 10^4^ HEK293 or HCT116 cells were seeded in the upper transwell chamber (24 inserts, 8 μm, Corning) coating with matrigel (Corning) in DMEM high glucose or DMEM-F12 and 0.01% BSA without FBS. DMEM supplemented with 10% FBS was used as chemoattractant in the lower chamber. After being incubated for 16 h, the invasive cells through the matrigel layer were fixed with formaldehyde, stained with *gelred* (Biotium) and counted. Images were captured by fluorescence microscope (Olympus BX51).

### Proliferation assay

The proliferation assay was performed as previously described [20]. Briefly 1 × 10^3^ cells were seeded onto 96 well plates. After 24 h, the medium was substituted by fresh medium in two different conditions: normal (DEMEM + 10% SFB) or low (DEMEM + 0.1% SFB) serum condition. The cell number was determined at 24, 48 and 72 h by spectrophotometry at 570 nm after staining with crystal violet.

### Hoechst efflux

Hoeschst efflux assay was performed as previously described [32], with some modifications. Briefly, cells were stained with 5 µg/ml Hoechst 33342 in presence or absence of 50 µM Verapamil (Sigma), for 90 minutes at 37°C. Then, the cells were washed and suspended in PBS. The Hoechst efflux in the presence or the absence of Verapamil was determined by spectrophotometry at 350 nm and cell number determined by counting under fluorescence microscopy.

### MMP activity assay

MMP2 enzymatic activity was determined by zymography, as previously described by [43]. Briefly, semiconfluent cell monolayers growing in 35-mm plates were extensively washed with PBS. Serum-free medium (1 ml) was added and incubation was continued for 24 h. Conditioned media containing MMP activity were harvested and frozen, while the remaining monolayers were lysed with 1% Triton X-100 in PBS, and cell protein content was determined (Bio-Rad Protein Assay). Samples were run on 9% SDS polyacrylamide slab gels containing 1 mg/ml of gelatin, under non-reducing conditions. After electrophoresis, gels were washed for 30 min using in 2.5% Triton X-100 and subsequently incubated for 48 h at 37°C in a buffer containing 0.25 M Tris-HCl pH 7.4, 1 M NaCl, and 25 mM CaCl_2_. Non-specific activity was detected using gels incubated in the same buffer solution but supplemented with 40 mM EDTA. After incubation, gels were fixed and stained with 2% Coomassie Brilliant Blue G-250 in methanol/acetic acid/H2O (30:10:60). The white bands corresponding to MMP2 activity were determined and analyzed using Image J program after Coomassie Blue (Sigma) staining, where the densitometry values for each band was relativized to the protein contents of conditioned medium as determined by Bradford assay.

### CD133-positive or depleted side population preparation

Side-populations were prepared following the instructions of the magnetic separation kit from Milteni company. Briefly, 2 × 10^8^ HCT116 cells in buffer (PBS + 0,5% BSA+2 mM EDTA) were mixed with 2 mg of anti CD133 antibody (AC133, Milteni) for 15 minutes, then, cells were centrifuged and resuspended in the same buffer and 15 ml of magnetic A/G beads were added and slowly mixed in orbital shaker for 30 minutes. The complexes were washed two times and centrifuged at 300 × g for 10 minutes and resuspended in buffer.

CD133+ and CD133-depleted side-populations were prepared by using of μMACS columns. The fraction obtained after two washes of cells bound to magnetic beads that were retained in the column; correspond to CD133+ side-population, while the CD133− side-population was collected from the excluded fraction after a second passage into the columns.

### Analysis of microarrays

The RAC3 expression was analyzed in pools of GEO (Gene Expression Omnibus) public repository microarrays data bank:

I) Platform GPL570 (Affymetrix Santa Clara, CA, USA), accession number GDS4718, Analysis of homogenized colorectal cancer (CRC) tumors representing various stages and metastases.
II) Platform GPL96 (Affymetrix Santa Clara, CA, USA), accession number GSE24747 for CaCo (colon cancer cells). The side population CD133+ o CD133− were isolated by flow cytometry.
III) Platform GPL6244 (Affymetrix Santa Clara, CA, USA), accession number GSM932995 for HCT116 (colon cancer cells). The side population CD133+ o CD133− were isolated by flow cytometry.

### *In vivo* experiments

Animal experiments were performed in accordance with Guide for Care and Use of Laboratory Animals (National Institute of Health, US), Britannic Committee research animals (United Kigdom Coordinating Committee on Cancer Research, 1998; Woekman *et al.*, 2010) and approved buy our Animal Ethics Committee.

Male Nude (*nu/nu*) mice, 7–10 weeks old, were housed in the animal facility of the School of Sciences, University of Buenos Aires with food and water *ad libitum*. Mice were randomly divided into two groups and inoculated subcutaneously at the right dorsal flank with 2 × 10^6^ of RAC3-HEK293 or EV-HEK293 transfected cells. No changes in weight were detected. Tumor size was calculated as *W*^2^ × *L*/2, where *W* = width and *L* = length. Tumor volume was normalized to tumor volume at the start day of exponential tumor growth [46].

Histopathology was performed in sections from two tumors by staining with hematoxylin and eosin after 20 days of inoculation. Furthermore, under our experimental conditions, the inoculated cells did not produce any toxic effect as animals gained weight as control animals, and the autopsy of animals did not reveal any signs of toxicity as confirmed by the pathologist.

### Statistics analysis

Each experiment was performed in triplicate and repeated at least three times. The differences in mean values among groups were evaluated and expressed as the mean ± SD. *T*-test were used for data analysis.

## Abbreviations

ABC transporters: ATP-binding cassette transporters
ABCG2: ATP-binding cassette sub-family G member 2
BSA: Bovine Serum Albumin
DMEM: Dulbecco’s Modified Eagle Medium
EV: Empty Vector
EGF: Epidermal growth factor
FBS: Fetal Bovine Serum
GAPDH: Glyceraldehyde 3-phosphate dehydrogenase
G418: Neomicyn
HEK293: Human embryonic kidney cells
HCT116: human colon cancer cells
MMP: Metalloproteinase
MDR: Multidrug Resistance
NF-ҡB: Nuclear factor kappa
OCT4: Octamer-binding transcription factor 4
shRAC3: Short harping for Receptor Associated Coactivator 3
TNF: Tumor necrosis factor
T47D: Human breast cancer cells
